# “Sentinel lymph node imaging with sequential SPECT/CT lymphoscintigraphy before and after neoadjuvant chemoradiotherapy in patients with cancer of the oesophagus or gastro-oesophageal junction – a pilot study”

**DOI:** 10.1186/s40644-018-0185-1

**Published:** 2018-12-18

**Authors:** Stefan Gabrielson, Jon A. Tsai, Fuat Celebioglu, Magnus Nilsson, Ioannis Rouvelas, Mats Lindblad, Annie Bjäreback, Artur Tomson, Rimma Axelsson

**Affiliations:** 1 Department of Nuclear Medicine, Karolinska University Hospital, C1-46, SE-141 86 Huddinge, Stockholm, Sweden; 2 Department of Clinical Science, Intervention and Technology, Division of Radiology, Karolinska Institutet, C1:46, Huddinge, S-141 86 Stockholm, Sweden; 3 Department of Clinical Science, Intervention and Technology, Division of Surgery, Karolinska Institutet, K53 Huddinge, S-141 86 Stockholm, Sweden; 4 Department of Clinical Science and Education, Södersjukhuset, Division of Surgery, Sjukhusbacken 10, 118 83 Stockholm, Sweden; 5 Department of Surgery, Södersjukhuset, 118 83 Stockholm, Sweden; 6 Department of upper abdominal diseases, Karolinska University Hospital, Karolinska University Hospital, Stockholm, Sweden

**Keywords:** Oesophageal cancer, Neoadjuvant therapy, Lymphatic structures, Sentinel lymph node concept, SPECT/CT

## Abstract

**Background:**

In current best practise, curatively intended treatment for oesophageal cancer usually consists of neoadjuvant chemo-radiotherapy (nCRT) or perioperative chemotherapy, and oesophagectomy. Sentinel Lymph Node Biopsy (SLNB) has the potential to identify patients without lymph node metastases and thus improve the staging accuracy and influence treatment. The impact of neoadjuvant treatment on the lymphatic drainage of oesophageal cancers and subsequently the SLNB procedure in this tumour type has previously not been well studied.

**Purpose:**

To evaluate changes in lymphatic drainage patterns of the tumour in patients with cancer of the oesophagus or gastro-oesophageal junction (GOJ) using Sentinel Lymph Node (SLN) hybrid SPECT/CT lymphoscintigraphy before and after nCRT.

**Methods:**

Patients with clinical stage T2-T3, any N-stage, M0 cancer of the oesophagus or GOJ underwent endoscopically guided peri−/intratumoral injection of radio-colloid followed by hybrid SPECT/CT lymphoscintigraphy prior to, and once again following, nCRT. SPECT/CT images were evaluated to number and location of SLNs and compared between the two examinations.

**Results:**

Ten patients were included in this pilot trial. SPECT/CT lymphoscintigraphy was performed in twenty procedures. The same Sentinel Lymph Node station before and after nCRT was observed in one single patient. In two patients, no SLN was detected before nCRT. In three patients no SLN was detected following nCRT. In four patients, the SLN stations were not the same station at baseline compared to follow-up examination.

**Conclusions:**

The reproducibility SLN detection in patients with cancer of the oesophagus/GOJ following nCRT was very poor. nCRT appears to alter lymphatic drainage patterns and thus may affect detection of SLNs and potentially also the accuracy of an SLNB in these patients. On the basis of these initial results, we abort further patient recruitment in our institution.

**Trial registration:**

Australian New Zealand Clinical Trials Registry (ANZCTR). Identifier ACTRN12618001433291. Date registered: 27/08/2018. Retrospectively registered.

## Background

Globally, cancer of the oesophagus or gastroesophageal junction is the ninth most common form of cancer [1]. The incidence of adenocarcinoma (AC) of the oesophagus or gastroesophageal junction is rising in the Western world, presumably due to increases in obesity and associated gastro-oesophageal reflux [2]. One of the most important prognostic factors in oesophageal cancer is the presence of lymphatic dissemination to loco-regional lymph nodes. Routinely, curatively intended treatment of most stage ≥T2, any N-stage oesophageal cancer consists of oesophagectomy in conjunction with a two-field lymphadenectomy. This procedure includes dissection of mediastinal and abdominal lymph nodes in well-defined lymph node stations [3].

The addition of neoadjuvant chemotherapy (nCT) or perioperative treatment with chemotherapy (pCT) or neoadjuvant chemo-radiotherapy (nCRT) has been shown to improve long-term survival in patients with cancer of the oesophagus or GOJ [4–6]. It is routinely used in patients with T2 or higher stage tumours prior to oesophagectomy.

Oesophagectomy with abdominal and mediastinal lymphadenectomy is an extensive surgical procedure which requires thoracoabdominal approach regardless of whether it is performed as an open or minimally invasive operation. Theoretically, the degree of lymphadenectomy could be reduced in selected patients if there were a reliable method for limited and targeted lymph node sampling.

The Sentinel Lymph Node Biopsy (SLNB) method is well established in the treatment of breast cancer and has been investigated in the setting of several cancers of the gastro-intestinal system [7]. Results of studies on the validity of the SLNB method in cancer of the oesophagus or GOJ have been encouraging. By using techniques primarily with radio-guided intraoperative Sentinel Lymph Node (SLN) identification, detection rates exceed 90% for all T-stages in adenocarcinoma as well as in squamous cell carcinoma (SCC) [8]. The sensitivity of the SLNB method varies in regard to T-stage, with better results for stage T1 cancers (91,7%), and unacceptable levels in stage T3 cancers (50%). The addition of preoperative SLN mapping with gamma camera lymphoscintigraphy has been shown to improve detection rates of SLNs [9–12]. By using Single Photon Emission Tomography (SPECT) combined with Computed Tomography (CT) in hybrid system (SPECT/CT lymphoscintigraphy), SLN detection rates have been even further improved in breast cancer patients compared to planar gamma camera imaging [13].

The impact of nCRT on the SLNB method in oesophageal cancer has been the subject of few studies and results have been conflicting with detection rates of 54%[29.1–77%] and sensitivity rates of 25% [1–81%] [8] in patients undergoing SLNB following neoadjuvant chemotherapy or chemo-radiotherapy.

Very few of those studies investigating the validity of the SLNB method in oesophageal cancer have included patients with a previous history of neoadjuvant treatment. Most studies have included a mixed population of patients with a significant minority of participants having been exposed to neoadjuvant treatment prior to surgery and SLNB. The SLNB method is well established in the staging of breast cancer, and the effects of neoadjuvant chemotherapy or neoadjuvant radiotherapy have been better studied. Neoadjuvant radiotherapy has been shown to significantly lower the detection rates of SLNs in breast cancer patients undergoing a SPECT/CT lymphoscintigraphy [14, 15]. Other studies have shown not only lower detection rates of SLNs in the same patient group, but also significantly lower accuracy of the SLNB procedure following nCT [16, 17].

Results from previous studies on the subject of the SLNB method in oesophageal cancer have been conflicting. Two studies have shown a tendency towards lower detection rates of SLNB in patients with previous history of nCT. On the other hand, other studies have not shown any significant differences in detection rates in patients with or without nCT [10] or nCRT [18].

Previous studies of the clinical effects of radiation therapy on lymphatic structures and function have been conflicting. One study of breast cancer patients following external beam radiotherapy showed a significant increase in lymphatic capillaries, albeit with no evidence of effect on the clinical function of lymphatic drainage of the same area [19]. Another study using a murine model showed that radiation doses of up to 30 Gy to the tail of the mouse resulted in decreases in lymphatic capillaries as well as a reduction of lymphatic function. [20]. In a rabbit model of the effects on lymphatic flow following 24 Gy radiation to a single lymph node, the authors observed a significant reduction of lymphatic flow [21].

The effects of neoadjuvant chemotherapy on the accuracy of SLNB are likewise not well studied. The underlying mechanisms by which alterations in lymphatic drainage following chemotherapy may occur and thus affect lymphatic mapping are poorly understood. Suggested factors include fibrosis of lymphatic structures, as well as lymphatic obstruction by cellular debris or tumour embolization.

As many patients with cancer of the oesophagus/GOJ where the SLNB method might be useful, will undergo neoadjuvant chemo-radiotherapy, it is important to better understand the effects of this treatment on the lymphatic drainage from the tumour and thereby the impact potential changes will have on the accuracy of a SLNB procedure.

### Aims

The aim of this study is to investigate the effect of neoadjuvant chemo-radiotherapy.

on tumour lymphatic drainage patterns in patients with cancer of the oesophagus or GOJ using sequential SPECT/CT lymphoscintigraphy before and following chemo-radiotherapy but before surgery.

## Methods

### Patients

Study participants were recruited prospectively between 2013 and 2015 at Karolinska University Hospital. Patients eligible for inclusion had to have histologically verified, clinical stage T2-T3, any N-stage, M0 cancer of the oesophagus or GE-junction planned for oesophagectomy following neoadjuvant chemo-radiotherapy. All patients were staged using endoscopically guided biopsies, contrast-enhanced Computed Tomography and ^18^F-FDG Positron Emission Tomography/Computed Tomography (PET/CT). Other inclusion criteria were patient age ≤ 75 years, physical performance status allowing oesophagectomy and with a performance status, renal and haematological status permitting chemotherapy.

### Neoadjuvant chemo-radiotherapy and surgery

All study participants were planned for neoadjuvant chemo-radiotherapy consisting of three cycles of Cisplatin/Oxaliplatin-5-FU and external beam radiation therapy with a total dose of 40 Gy given in fractions.

### SPECT/CT lymphoscintigraphy

Shortly prior to neoadjuvant chemo-radiotherapy, patients underwent endoscopic submucosal radio colloid injection of 4 X 0.5 mL in total of 60 MBq 99mTc-nanocoll (GE Healthcare Srl., Milan, Italy) peri- and intratumorally. Whole body planar gamma camera imaging was performed 1 h after radio colloid injection in order first to localize the sentinel node (SN), (256 X 1024, 10 cm/min). This image was used to centre the SPECT/CT examination. A Siemens Symbia T16 (Symbiaw Siemens, Erlangen, Germany) with a low energy, high-resolution collimator was used for all imaging.

SPECT imaging was performed using a 128X128 matrix, 64 projections over 360° and 40 s per projection. CT scan of the same anatomical region with 110 kV, 75mAs and pitch 1.3. Iterative reconstruction of the SPECT data was done with OSEM, four iterations, eight subsets including resolution recovery. A gaussian postfiltration was applied with 0.75 cm FWHM.

The same procedure was repeated following the conclusion of neoadjuvant chemo-radiotherapy and preceding oesophagectomy.

### Image evaluation

**Fig. 1:**
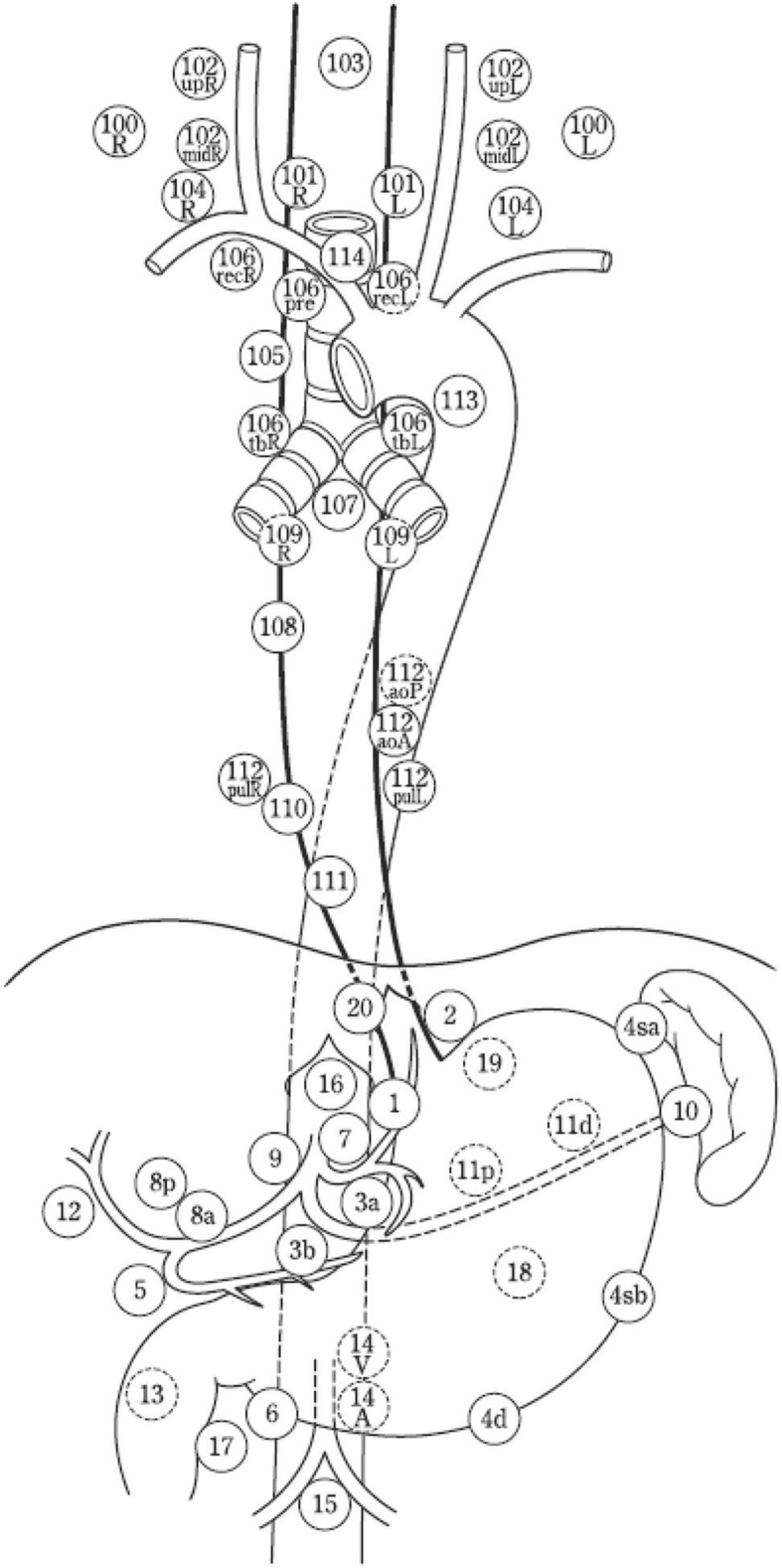
Lymph node stations as per the Japanese Classification of Oesophageal Cancer, 11th Edition: part I. ©, as well as credit to the original authors. This image is reproduced in an unaltered state under the terms of the Creative Commons Attribution 4.0 International License (http://creativecommons.org/licenses/by/4.0/)

All image reconstructions and image evaluation were made using Hermes Hybrid viewer (Hermes Medical solutions, Stockholm, Sweden). Using this software, transverse SPECT images were fused with transverse CT images (0.75 mm/ 0.7 mm recon increment, B31 medium smooth kernel). Multiplanar reconstruction was performed with resulting images in transverse, coronal, and sagittal planes. In SPECT images, sites of injection were masked with hand-drawn volumes of interest (VOIs) in order to accentuate the uptake in SLNs. Any discernible radio-colloid uptake/uptakes in SPECT images with corresponding lymph nodes on CT was considered a Sentinel Lymph Node. SLN stations were classified in accordance with the Japanese Classification of oesophageal cancer 11th edition (distribution of lymph node stations is illustrated in Fig. 1) [22].

Sentinel Lymph Node uptake was compared in the same patients in SPECT/CT before and after nCRT in regard to number and location(s). SPECT/CT images were evaluated by either SG or RA.

## Results

**Fig. 2:**
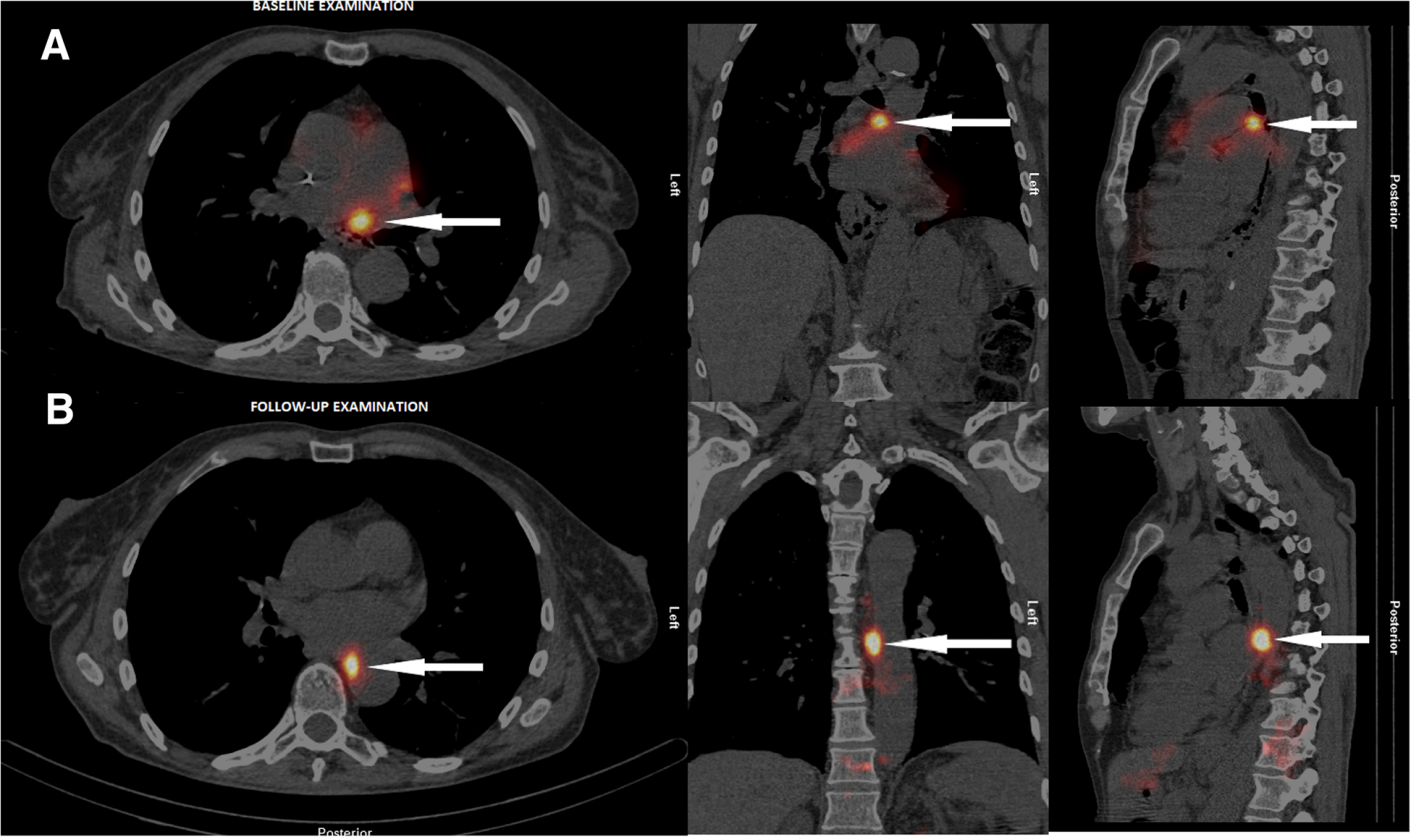
Hybrid SPECT/CT images of a 68-year-old female patient with a cT2 N0 M0 adenocarcinoma located in the cardia in axial, coronal and sagittal views from left to right in **a**) Baseline examination, **b**) Follow-up examination. White arrows indicate radio-colloid uptake. In the baseline examination a SLN was located in the 112 station. In the follow-up examination a SLN was instead located in the 109 L station

Ten patients were included in this pilot trial. Eight out of the ten patients underwent three cycles of Cisplatin/Oxaliplatin-5-FU. One patient underwent two cycles due to hyperemesis and one patient underwent only one cycle due to kidney failure. All ten patients underwent radiation therapy with a total dose of 40 Gy. Patient and tumour characteristics are presented in Table 1. Hybrid SPECT/CT lymphoscintigraphy prior to and following nCRT was performed on all ten patients. At baseline examination, the median number of identified SLN stations was 1 (range 0–2). In two patients, no SLN was identified at baseline examinations. At follow-up examination, the median number of identified SLN stations was also 1 (range 0–1). In three patients, no SLN was identified at follow-up examinations. Distributions of SLN stations at baseline and follow-up examinations are presented in Table 2. SPECT/CT lymphoscintigraphy of one patient at baseline examination and at follow-up examination is illustrated in Fig. 2.

**Table 1:** Patient and tumour characteristics

Patient id	Age/Sex	Tumour type	Tumour location^a^	cTNM-stage^b^	pTNM-stage^c^	No. Lymph Nodes	No. Metastatic Lymph Nodes
1	M/69	Adenocarcinoma	Cardia Siewert 2	T3N2M0	T2N2M0	36	0
2	M/70	Adenocarcinoma	Cardia Siewert 2–3	T3N1M0	T3N0M0	11	0
3	M/64	Adenocarcinoma	Cardia Siewert 2	T3N0M0	T1N3M0	38	9
4	F/68	Adenocarcinoma	Cardia Siewert 2	T3N0M0	T2N0M0	27	0
5	M/65	Adenocarcinoma	Cardia Siewert 1–2	T3N1M0	T1aN0M0	22	0
6	M/64	Adenocarcinoma	Cardia Siewert 1	T3N0M0	T0N0M0	30	0
7	M/63	Adenocarcinoma	Cardia Siewert 2	T3N2M0	T3N3M0	33	20
8	M/60	Squamous cell carcinoma	Distal third/ cardia	T3N0M0	T1N0M0	11	0
9	M/79	Squamous cell carcinoma	Middle third	T2N1M0	T0N0M0	22	0
10	M/54	Adenocarcinoma	Cardia Siewert 1–2	T3N1M0	T3N0M0	9	0

^a^Tumour location according to the Siewert classification

^b^Clinical TNM-stage at time of diagnosis

^c^Pathological TNM-stage

**Table 2:** SLN detection and distribution of SLN stations

Patient id	Age/sex	Tumour type	Baseline SLN stations (N)	Baseline SLN station locations	Follow-up SLN stations (N)	Follow-up SLN station locations
1	M/69	Adenocarcinoma	2	111,112	0	NA
2	M/70	Adenocarcinoma	2	111,2	1	16a2
3	M/64	Adenocarcinoma	1	110	1	1
4	F/68	Adenocarcinoma	1	112	2	109 L,110
5	M/65	Adenocarcinoma	1	3	0	NA
6	M/64	Adenocarcinoma	0	NA	1	1
7	M/63	Adenocarcinoma	0	NA	2	107,109R
8	M/60	Squamous cell carcinoma	1	108	0	NA
9	M/79	Squamous cell carcinoma	1	101 L	1	101 L
10	M/54	Adenocarcinoma	2	109 L (2)	2	105,111

Four to six weeks following neoadjuvant treatment, all patients underwent surgery with open oesophagectomy and two field lymph node dissection.

## Discussion

This study shows that the reproducibility of SPECT/CT lymphoscintigraphy following neoadjuvant chemo-radiotherapy was very poor. In only one case (Patient No. 9), out of the ten studied, was the same SLN station identified in examinations before and after neoadjuvant chemo-radiotherapy. In three cases where at least one SLN station was detected at baseline, there was neither any discernible radio-colloid uptake in the same stations at follow-up nor were there any other detectable SLN stations. In two patients where no SLNs could be detected at baseline, at least one SLN station was detected at follow-up examination. Out of the five patients where SLN stations could be detected at both examinations, the SLN stations were not the same at follow-up compared to baseline examination in four cases.

Due to the low frequency of patients with positive lymph nodes at final pathological diagnosis, we were unable to correlate any changes in SLN distribution to the histopathological data.

The impact and underlying mechanisms of neoadjuvant treatment on lymphatic drainage patterns in oesophageal cancer is not well studied. The present study suggests that neoadjuvant chemo-radiotherapy may result in significant changes in the lymphatic drainage patterns. Such changes may prove detrimental to the accuracy of the SLNB method in this patient group and may explain the poor accuracy of SLNB in patients who have received neoadjuvant therapy in previous investigations [11, 23].

It is interesting to note that the only reproducible SLN lymphoscintigraphy in this pilot trial was in the one patient with clinical T2 stage tumour, whereas all other patients were staged as T3. It is also noteworthy that this patient was one of two patients with squamous cell carcinoma located in the mid-oesophagus with the SLN station being located in the cervical region (station 109 L). The significance of these characteristics is, however, unclear.

The fact that we were unable to detect a SLN at baseline examination in 2/10 cases is in line with results in previous studies of intra-operative radio-guided SLNB with detection rates of 77.5% [57.4–89.8%] in T3-T4 stage tumours [8].

As there are no previous studies specifically addressing the effects of chemo-radiotherapy on changes in lymphatic drainage in the oesophagus, the results presented here must instead be compared to other cancers, mainly breast cancer, where Sentinel Lymph Node imaging has been applied in patients being exposed to radiation or chemotherapy. For example, in 2009, van der Ploeg et al. published results from a study of 22 patients with breast cancer previously treated with mantle-radiation due to Hodgkin’s Lymphoma, undergoing SLN mapping with SPECT/CT lymphoscintigraphy and radio−/blue dye guided SLNB. Failure to identify SLN was significantly higher in the treated group compared to the control group (14 and 3% respectively (*P* = 0.01)). Moreover, there were significantly more patients in which the SLN was identified outside of the axilla, compared to a treatment naïve control population (41 and 33% respectively (*P* = 0.04)) [14].

The same investigators also reported on the reliability of SLNB in patients with recurrent breast cancer. In order to improve SLN detection, preoperative SPECT/CT lymphoscintigraphy was performed [15]. 36 of 114 (31%) patients included in this study had undergone breast-preserving therapy including radiotherapy without axillary lymph node dissection. The authors found that the detection rates were significantly lower in patients with a history of breast-conserving therapy, compared to the whole study population, with detection rates of 72% compared to 85% for the latter (*P* = 0.01). In the same sub-group, the SLN was more often located outside the axilla adjacent to the primary tumour, often as far away as in the contra-lateral axilla. It is, however, difficult to assess in what order of magnitude radiotherapy to the breast, radiation to the lymphatics in the axilla and variation in surgical technique when excising the primary tumour will affect the flow of lymphatics from the breast cancer to the Sentinel Node(s). The authors conclude that the SLNB method is probably more reliable in the setting where no iatrogenic disturbance of the lymphatics has occurred.

Another cancer where the SLNB method is under investigation is prostate cancer. A pilot study of ten patients with relapse following previous treatment with either external beam radiation, brachytherapy or high intensity focused ultrasound was conducted in 2010. The authors found that at least one SLN could be identified in all ten cases using SPECT/CT lymphoscintigraphy following intra-prostate injection of radio colloid. However, a much higher proportion of treated patients had a SLN outside of the pelvic parailiacal lymph node stations when compared to a group of 70 treatment naïve patients (80% compared to 34% (*P* = 0.01)) [24].

In 2013 Kuehn et al. published results of a large multi-centre cohort study of women with clinically node-negative breast cancer undergoing SLNB prior to chemotherapy [16]. The detection rate and accuracy of the SLNB was excellent, and at least one SLN was identified in of 1013/1022 patients (99.1%). In a subgroup of SNLB-negative patients undergoing a second SLNB following nCT, the detection rate was much lower with successful SLNB in only 213/360 patients (60.8%). Likewise, the false negative rate for SLNB was high in this second procedure, with a false negative SLNB in 33/64 patients (51.6%). Similar results were found in a recent study of the accuracy of SLNB performed prior to neoadjuvant chemotherapy in 224 breast cancer patients. The detection rate was 100 % with at least one SLN identified using established techniques for intraoperative SLN detection [17]. Approximately half of the patients in this study (98 patients) who were SNLB-negative underwent a second SLNB procedure following nCT, and the success rate of SNL detection was 69.4%. The false negative rates in SLNB were also significantly higher in repeated procedures group (25% compared to 7.4%). In conclusion, the authors do not recommend a second SLNB following nCT due to low SLN detection rates and unacceptable levels of false negative SLNBs.

To our knowledge, the present study is the first dedicated publication on sequential SPECT/CT lymphoscintigraphy in patients with cancer of the oesophagus or GOJ undergoing neoadjuvant therapy. Due to the limited sample size, our results must be interpreted with caution. Our results are in line with some, but not all, previous studies concerning detection rates of Sentinel Lymph Nodes in patients with cancer of the oesophagus/GOJ following neoadjuvant chemo-radiotherapy [8]. As there are no previous studies on the reproducibility of preoperative SLN mapping either in healthy subjects or in patients without a history of neoadjuvant treatment certain methodological aspects must be considered. Since the two procedures were conducted with a considerable interval, it is possible that differences in localization and depth of radiocolloid injection could influence the Sentinel Lymph Node in a second procedure. In the absence of dedicated studies, the accuracy of the SLNB-method in stage T1-tumors is high (96,1%) [12]. This may indicate that the reproducibility of preoperative SLN-mapping would be good in early oesophageal cancer, in the absence of neoadjuvant therapy. Considering the small number of patients in this study it is possible that future research using the same methodology on a larger patient population could yield valuable histopathological data on nodal metastatic status. This data could be used to correlate changes in lymphatic drainage patterns in SPECT/CT examinations.

## Conclusions

In this small pilot study, we observed very poor reproducibility in SLN detection in patients with oesophageal cancer before and after nCRT. We conclude that nCRT may have a clinically relevant impact on lymphatic drainage in the vast majority of these patients. We suggest that this may negatively influence the accuracy of a SLNB procedure in the same way as demonstrated in other cancer forms.

## Abbreviations

^18^F-FDG: Flourodeoxyglucose
^99^Tc: ^99m^Technetium
AC: Adenocarcinoma
CT: Computed tomography
FWHM: Full width at half maximum
GOJ: Gastro-esophageal Junction
Gy: Gray
kV: Kilovolt
mAs: Milliampere-second
MBq: Mega-becquerel
nCRT: Neoadjuvant chemo-radiotherapy
nCT: Neoadjuvant chemotherapy
OSEM: Ordered subset expectation maximization
pCT: Perioperative chemotherapy
SCC: Squamous cell carcinoma
SLN: Sentinel lymph node
SLNB: Sentinel lymph node biopsy
SPECT: Single Photon Emission Tomography
VOI: Volume of interest
